# Supplementary material for: Conventional and Deep-Learning-Based Image Reconstructions of Undersampled K-Space Data of the Lumbar Spine Using Compressed Sensing in MRI: A Comparative Study on 20 Subjects
Source: Diagnostics (Basel). 2023 Jan 23;13(3):418. doi: 10.3390/diagnostics13030418 (PMC9914543; doi:10.3390/diagnostics13030418)
Supplement: Supplementary file 1 [file diagnostics-13-00418-s001.zip › diagnostics-2117693-supplementary.pdf]

diagnostics

MDPI

Supplementary Materials

Scheme S1. a. Results of subjective reading: level of significance in the comparison of the delimitability of the bone marrow. Abbreviations: CS, conventional reconstructions of Compressed SENSE images; CS AI = deep-learning-based reconstructions of Compressed SENSE images, n.s. = not significant

	Standard	CS 4.5	CS AI 4.5	CS 8	CS AI 8	CS 11	CS AI 11
**Standard**	-	$p = 0.0443$	p=0.0007	n.s.	n.s.	n.s.	n.s.
**CS 4.5**		-	n. s.	$p = 0.0093$	n. s.	$p < 0.0001$	n.s.
**CS AI 4.5**			-	$p = 0.0001$	n. s.	$p < 0.0001$	$p = 0.0081$
**CS 8**				-	$p = 0.0208$	n. s.	n.s.
**CS AI 8**					-	$p < 0.0001$	n.s.
**CS 11**						-	$p = 0.0160$
**CS AI 11**							-

Scheme S1. b. Results of subjective reading: level of significance in the comparison of the delimitability of the intervertebral disc. Abbreviations: CS = conventional reconstructions of Compressed SENSE images, CS AI = deep-learning-based reconstructions of Compressed SENSE images, n. s. = not significant.

	Standard	CS 4.5	CS AI 4.5	CS 8	CS AI 8	CS 11	CS AI 11
**Standard**	-	n.s.	$p = 0.0016$	n.s.	n.s.	n.s.	n.s.
**CS 4.5**		-	n.s.	n.s.	n.s.	n.s.	n.s.
**CS AI 4.5**			-	$p < 0.0001$	p<0.0001	n.s.	$p = 0.0236$
**CS 8**				-	n.s.	$p = 0.0005$	n.s.
**CS AI 8**					-	$p = 0.0005$	n.s.
**CS 11**						-	n.s.
**CS AI 11**							-

Scheme S1. c. Results of subjective reading: level of significance in the comparison of the delimitability of the spinal cord. Abbreviations: CS = conventional reconstructions of Compressed SENSE images, CS AI = deep-learning-based reconstructions of Compressed SENSE images, n.s. = not significant.

	Standard	CS 4.5	CS AI 4.5	CS 8	CS AI 8	CS 11	CS AI 11
**Standard**	-	n. s.	$p = 0.0046$	n. s.	n. s.	n. s.	n. s.
**CS 4.5**		-	n. s.	n. s.	n. s.	$p = 0.0093$	n. s.
**CS AI 4.5**			-	$p = 0.0002$	n. s.	$p < 0.0001$	$p = 0.0016$
**CS 8**				-	$p = 0.0070$	n. s.	n. s.
**CS AI 8**					-	$p = 0.0010$	$p = 0.0392$
**CS 11**						-	n. s.
**CS AI 11**							-

Scheme S1. d. Results of subjective reading: level of significance in the comparison of the delimitability of the cerebrospinal fluid. Abbreviations: CS = conventional reconstructions of Compressed SENSE images, CS AI = deep-learning-based reconstructions of Compressed SENSE images, n.s. = not significant.

	Standard	CS 4.5	CS AI 4.5	CS 8	CS AI 8	CS 11	CS AI 11
**Standard**	-	n.s.	n.s.	n.s.	n.s.	n.s.	n.s.
**CS 4.5**		-	n.s.	n.s.	n.s.	$p = 0.0016$	n.s.
**CS AI 4.5**			-	$p = 0.0160$	n.s.	$p = 0.0003$	n.s.
**CS 8**				-	$p = 0.0070$	n.s.	n.s.

**CS AI 8**					-	$p = 0.0001$	n.s.
**CS 11**						-	n.s.
**CS AI 11**							-

Scheme S1. e. Results of subjective reading: level of significance in the comparison of the delimitability of the nerve roots. Abbreviations: CS = conventional reconstructions of Compressed SENSE images, CS AI = deep-learning-based reconstructions of Compressed SENSE images, n.s. = not significant.

	**Standard**	**CS 4.5**	**CS AI 4.5**	**CS 8**	**CS AI 8**	**CS 11**	**CS AI 11**
**Standard**	-	n.s.	n.s.	n.s.	n.s.	$p = 0.0010$	n.s.
**CS 4.5**		-	n.s.	n.s.	n.s.	$p = 0.0392$	n.s.
**CS AI 4.5**			-	n.s.	n.s.	$p = 0.0014$	n.s.
**CS 8**				-	n.s.	n.s.	n.s.
**CS AI 8**					-	$p = 0.0046$	n.s.
**CS 11**						-	n.s.
**CS AI 11**							-

Scheme S1. f. Results of subjective reading: level of significance in the comparison of the delimitability of the neuroforamina. Abbreviations: CS = conventional reconstructions of Compressed SENSE images, CS AI = deep-learning-based reconstructions of Compressed SENSE images, n.s. = not significant.

	**Standard**	**CS 4.5**	**CS AI 4.5**	**CS 8**	**CS AI 8**	**CS 11**	**CS AI 11**
**Standard**	-	n.s.	n.s.	n.s.	n.s.	$p = 0.0305$	n.s.
**CS 4.5**		-	n.s.	n.s.	n.s.	$p = 0.0009$	n.s.
**CS AI 4.5**			-	$p = 0.0208$	n.s.	$p = 0.0002$	n.s.
**CS 8**				-	n.s.	n.s.	n.s.
**CS AI 8**					-	$p = 0.0022$	n.s.
**CS 11**						-	n.s.
**CS AI 11**							-

Scheme S1. g. Results of subjective reading: level of significance in the comparison of overall impression. Abbreviations: CS = conventional reconstructions of Compressed SENSE images, CS AI = deep-learning-based reconstructions of Compressed SENSE images, n.s. = not significant.

	**Standard**	**CS 4.5**	**CS AI 4.5**	**CS 8**	**CS AI 8**	**CS 11**	**CS AI 11**
**Standard**	-	n.s.	n.s.	n.s.	n.s.	n.s.	n.s.
**CS 4.5**		-	n.s.	n.s.	n.s.	$p < 0.0001$	n.s.
**CS AI 4.5**			-	$p = 0.0107$	n.s.	$p < 0.0001$	$p = 0.0236$
**CS 8**				-	n.s.	n.s.	n.s.
**CS AI 8**					-	$p < 0.0001$	n.s.
**CS 11**						-	n.s.
**CS AI 11**							-
